# Signal Transducer and Activator of Transcription Factor 6 Signaling Contributes to Control Host Lung Pathology but Favors Susceptibility against *Toxocara canis* Infection

**DOI:** 10.1155/2013/696343

**Published:** 2013-01-30

**Authors:** Berenice Faz-López, Yadira Ledesma-Soto, Yolanda Romero-Sánchez, Elsa Calleja, Pablo Martínez-Labat, Luis I. Terrazas

**Affiliations:** ^1^Unidad de Biomedicina, Facultad de Estudios Superiores Iztacala, Universidad Nacional Autónoma de México (UNAM), Avenue de los Barrios 1, Los Reyes Iztacala, Tlalnepantla 54090, MEX, Mexico City, DE, Mexico; ^2^Laboratorio de Parasitología, Facultad de Estudios Superiores Cuautitlán, UNAM, 54714 Mexico City, DE, Mexico; ^3^Laboratorio de Histopatología, Facultad de Estudios Superiores Cuautitlán, UNAM, 54714 Mexico City, DE, Mexico; ^4^Carrera de Medicina, Facultad de Estudios Superiores Iztacala, UNAM, 54090 Mexico City, DE, Mexico

## Abstract

Using STAT6^−/−^ BALB/c mice, we have analyzed the role of STAT6-induced Th2 response in determining the outcome of experimental toxocariasis caused by embryonated eggs of the helminth parasite *Toxocara canis*. Following *T. canis* infection wild-type BALB/c mice developed a strong Th2-like response, produced high levels of IgG1, IgE, and IL-4, recruited alternatively activated macrophages, and displayed a moderate pathology in the lungs; however, they harbored heavy parasite loads in different tissues. In contrast, similarly infected STAT6^−/−^ BALB/c mice mounted a weak Th2-like response, did not recruit alternatively activated macrophages, displayed a severe pathology in the lungs, but efficiently controlled *T. canis* infection. These findings demonstrate that Th2-like response induced via STAT6-mediated signaling pathway mediates susceptibility to larval stage of *T. canis*. Furthermore, they also indicate that unlike most gastrointestinal helminths, immunity against larvae of *T. canis* is not mediated by a Th2-dominant response.

## 1. Introduction

Toxocariasis is a helminth infection considered as a zoonosis and is caused by the larvae of *Toxocara canis; *this parasite affects many paratenic hosts including humans. This infection is world-wide distributed mainly because its transmission is associated with domestic dogs. This disease is starting to be considered as a public health problem in Latin America and Asia [1, 2] but has been extended in the last few years to developed countries as shown by increased numbers of cases-report published more frequently [3–5]. Toxocariasis in humans results from accidental ingestion of *Toxocara-*embryonated eggs from excreta in the environment or by direct dog-to-person contact [6, 7]. Although larvae in muscle may be relatively symptom less, those in brain may cause serious neurological disorders [8]; these symptoms are believed to result from inflammation following degeneration of the parasite [9].

In the experimental model of murine toxocariasis, infection of inbred mice with *T. canis* induces a strong Th2-like response similar to that observed following infection with other helminthes such as *Nippostrongylus brasiliensis* and *Trichuris muris* [10–12]. While widely accepted, Th2-type response in clearing helminth infections has limitations [11, 13–17], its role in mediating protection against toxocariasis in paratenic hosts is not clear, mainly because reinfection in toxocariasis favors parasite survival [18]. Recent advances in the immunobiology of *T. canis* indicate that regulatory mechanisms are raised after infection; mainly T regulatory cells have been involved in limiting pathologic damage by the inflammatory response [19]; however, there are few data regarding on mechanism of protection or susceptibility against such parasite.

Previous studies have found that some extraintestinal larvae from other helminthes such as *Taenia crassiceps and Trichinella spiralis *are apparently eliminated in infected mice by a Th1-mediated inflammatory response during early phase of infection [20–22]. Furthermore, both studies found that STAT6 was involved during early phase of infection in rendering them more susceptible to cysticercosis and trichinellosis, respectively. These findings suggest that while Th2-type response may be involved in mediating resistance on gastrointestinal helminth infections, this pathway may be involved in susceptibility to the larval stages of such parasites. Numerous studies using STAT6^−/−^ mice have shown that STAT6-mediated IL-4/IL-13 signaling pathway is critical for Th2 differentiation [23]. For example, STAT6^−/−^ mice fail to mount a significant Th2 response and cannot control worm burdens following infection with gastrointestinal helminth parasites [17, 24]. Conversely, STAT6^−/−^ mice develop a Th1 like response and control infections caused by intracellular protozoan parasites such as *Leishmania mexicana* and *Trypanosoma cruzi* [25, 26] indicating that STAT6-mediated signaling pathway inhibits development of protective immunity by inhibiting a Th1 development. 

The purpose of this study was to determine the role of a Th2-type response induced via STAT6-mediated signaling in the outcome of experimental murine toxocariasis caused by the L2 of the nematode *T. canis*. To approach this question, we compared the course of *T. canis* infection in STAT6^−/−^ BALB/c mice (STAT6^−/−^) with that in the wild-type BALB/c (STAT6^+/+^) mice. In addition, we analyzed both the antibody and cytokine profiles in sera, as well as the phenotype of lung macrophages. Our data demonstrate that Th2-type response induced via STAT6-signaling pathway mediates susceptibility in toxocariasis. 

## 2. Materials and Methods

### 2.1. Mice

Six-8-week-old male STAT6^−/−^ and STAT6^+/+^ mice in a genetic BALB/c background were originally purchased from The Jackson Laboratory Animal Resources Center (Bar Harbor, Maine, USA) and were maintained in a pathogen-free environment at the FES-Iztacala, U.N.A.M. animal facility in accordance with Institutional and National guidelines. 

### 2.2. Isolation of Larvae and Eggs and Infection Protocol

Adult *T. canis* females' worms were isolated from the intestine of naturally infected puppies (<3 months). Isolation and embryonation of eggs were performed as follows: female worms were dissected and from the uterus eggs were isolated and putted into distilled water; then the mixture was centrifuged two times for 10 min at 2,000 ×g in a solution of NaHCl at 1%. After removal of the supernatant, the sediment was two times washed in distilled water and placed into the solution of formalin at 1% in tissue flasks at 28°C for 1 month with gentle daily agitation until the end of embryonation which was controlled under the microscope. 

### 2.3. Infection

Five hundred larvated eggs were intragastrically administered with a Foley tube to both STAT6^−/−^ and STAT6^+/+^ mice. Infected mice were sacrificed at days 5, 14, and 60 postinfection, and the parasites harvested from different tissues (lung, liver, brain, and muscle) were enumerated as described previously [27]. 

For histological evaluation of different tissues, animals were euthanized at indicated days. The liver, lung, brain, and muscle were removed and fixed in 4% formalin. Tissue samples were embedded in paraffin, and 5 *μ*m sections were cut on a microtome and stained with hematoxylin and eosin for histological examination. 

### 2.4. Cytokine Measurements

The IL-4, IL-12, and IFN-*γ* levels were quantified in mouse serum at the indicated point times. Antibody pairs were used according to the manufacturer's instruction (Peprotech México, México, DF).

### 2.5. * Toxocara-*Specific Antibody Level and Total IgE

Peripheral blood was collected at the indicated time points after Toxocara infection from tail snips. The blood was centrifuged at 2500 rpm for 10 min, and serum was collected and tested for Toxocara-specific IgG1 and IgG2a in antigen-coated plates (1 *μ*g/mL). After an overnight incubation at 4°C, the plates were washed with PBS supplemented with 0.05% Tween 20 (Sigma, St. Louis, MO, USA) and blocked with PBS supplemented with 1% BSA (US Biological, Swampscott, MA, USA). Serial dilutions (starting from 1 : 100) of the serum samples were added to the plates. The bound antibodies were detected following incubation with HRP-conjugated rat anti-mouse IgG1 or IgG2a (Zymed, San Francisco, CA, USA). The reactions were developed with ABTS solution (Zymed) and read on a microplate reader at 405 nm (Multiskan Ascent, Thermo Labsystems). Results are expressed as the maximal sera dilution (endpoint titer) where OD was detected. Total IgE production was detected by Opt-ELISA from Biolegend.

### 2.6. Flow Cytometry

It has been previously shown that macrophages recruited by helminth parasites to the site of infection express alternatively activated and suppressive markers, such as mannose receptor (MR), PD1 ligand 1 (PD-L1), and PD-L2. To determine whether *T. canis*-infected mice recruit such population, flow cytometry was performed on lung exudates cells from *T. canis*-infected at different times after infection. Briefly, 5, 14, and 60 days after infection, lungs lavages were aseptically obtained, and 1 × 10^6^ cells were incubated with anti-CD16 and anti-CD32 (Biolegend, San Diego, CA, USA) to block nonspecific antibody binding. The cells were then stained with APC-conjugated anti-F4/80, FITC-conjugated anti-MR, PE-conjugated anti-PDL1, and PE-conjugated anti-Gr1 (all from Biolegend) and incubated for 30 min. at 4°C in FACS staining buffer (1% FBS, 0.5% sodium azide in PBS). The cells were analyzed using a FACSCalibur and Cell Quest software (Becton Dickinson). 

### 2.7. RT-PCR

RNA was extracted from isolated spleen cells after different day's postinfection using the TRIzol Reagent (Invitrogen, Carlsbad, CA, USA) and the isopropanol-chloroform technique. The RNA was quantified, and 5 *μ*g of RNA was reverse transcribed using the Superscript II First Strand Synthesis Kit (Invitrogen). PCR reactions containing 5x PCR Buffer blue, 10 mM dNTP, 40 nM each forward and reverse primers, 1 U Taq DNA polymerase (Sacace Biotechnologies, Italy), and 2 *μ*L of the cDNA were prepared in a 25 *μ*L final volume. The PCR conditions were elsewhere described [28]. Briefly, consisted of an initial denaturation step at 95°C for 5 min; 35 cycles of 95°C for 40 s, the indicated melting temperature for 50 s and 72°C for 40 s; a final extension step at 72°C for 4 min in a thermal cycler (Corbett Research, Australia). The amplified products were mixed with loading buffer containing SYBR green and observed in a 1.5% agarose gel with the Fujifilm FLA 5000 scanner (Fuji, Japan) using the image reader V2.1 software to capture the images.

### 2.8. Statistical Analysis

Comparisons between wild-type (STAT6^+/+^) and STAT6^−/−^ groups considered in this work were made using student's unpaired* t* test. *P* < 0.05 was considered significant. The statistical significance of the sera titers were determined by nonparametric tests using Mann-Whitney U-Wilcoxon Rank.

## 3. Results and Discussion

**Figure 1:**
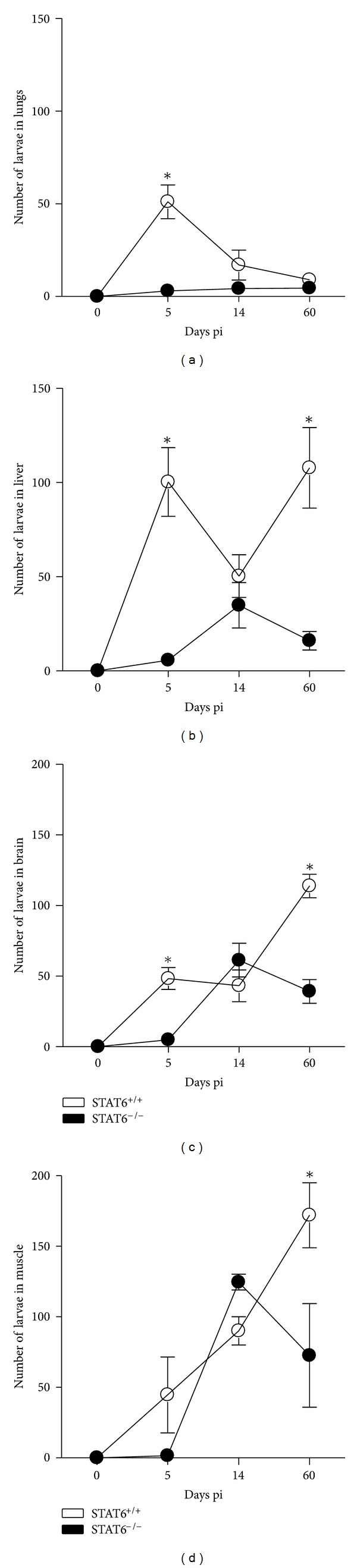
STAT6^−/−^ mice efficiently control *Toxocara canis* infection. Course of i.p. *T. canis* infection in STAT6^−/−^ (solid circles) and STAT6^+/+^ (open circles) mice following infection with 500 L2. Data are expressed as the mean ± SE of 4–6 mice per group. *P < 0.05 comparing STAT6^−/−^ versus STAT6^+/+^ at the same time point. Similar results were observed in two independent experiments.

**Figure 2:**
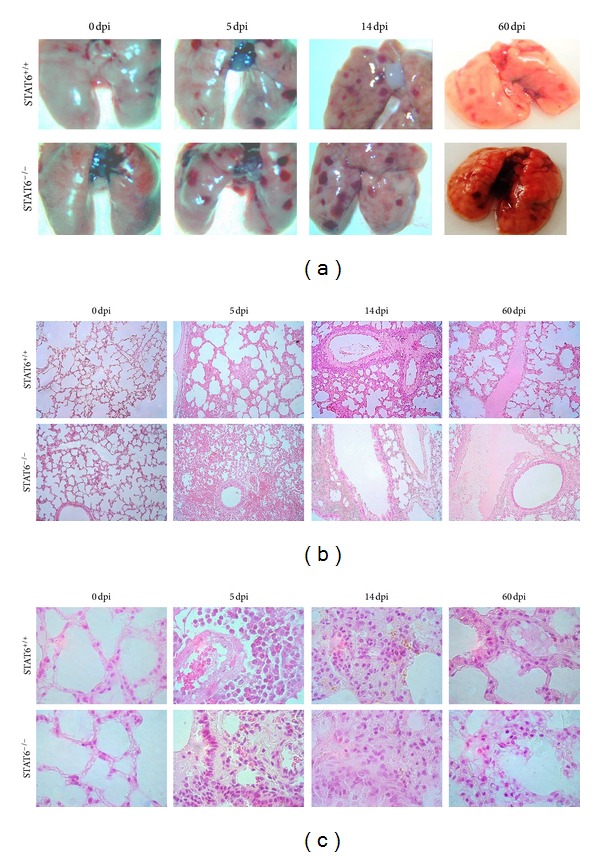
STAT6^−/−^ mice display a more severe pathology early in the infection with *T. canis*. (a) Macroscopic appearance of lungs obtained at different time points after oral infection with 500 Larvae of *T. canis*. (b) Lung histology showing airway inflammation in both groups. Magnification 40X. (c) Lung histology, 100X magnification.

It is largely accepted that the Th2-like response induced via STAT6-mediated signaling pathway (through IL-4/IL-13 receptors) plays a critical role in mediating protective immunity against most helminthes [12, 17, 28]. For example, STAT6-mediated signaling has been shown to promote protective immunity against gastrointestinal helminthes such as *Trichinella spiralis*, *N. brasiliensis,* and *Hymenolepis diminuta* [12, 17, 29, 30]. However, the role for many molecules associated with the immune response, including STAT6, during infection with *Toxocara canis* is unknown. Here we analyzed the potential role of STAT6 in modulating immunity against this nematode parasite. One of the first organs that *T. canis* larvae reach early after infection is the lungs, in the present study we detected a significant greater number of larvae in STAT6^+/+^ compared to STAT6^−/−^ mice at day 5 after infection, but later both groups displayed comparable parasite burdens at 14 and 60 days postinfection (Figure 1(a)). In contrast, in the liver as early as 5 days pi STAT6^−/−^ displayed lower parasite burdens, and this was more evident after 60 days pi (Figure 1(b)). In a similar way the parasite burdens in the brain were statistically different 60 days pi (Figure 1(c)). Interestingly, as infection progressed, the number of larvae in the muscles increased significantly in STAT6^+/+^ mice as compared to STAT6^−/−^ mice that successfully reduced the number of parasites by day 60 postinfection (Figure 1(d); *P < 0.01). Unexpectedly, the lungs from STAT6^−/−^ mice displayed a greater macroscopic damage as we observed an increased number of hemorrhagic spots in such organs (Figure 2(a)) although that numbers of parasites detected in both groups were closely similar at 14 days pi or even lower in STAT6^−/−^ mice at 5 days pi. It is known that *T. canis* infection promotes the recruitment of leukocytes to the lungs generating an acute inflammatory response; here we observed a greater inflammatory infiltration in the lungs of STAT6^−/−^ mice as early as 5 dpi which was maintained higher in these mice until 60 dpi (Figure 2(b)). Moreover, disruption of alveoli was more frequently observed in STAT6^−/−^ mice, as well as a dominant polymorphic cell infiltration (Figure 2(c)). These findings suggest that STAT6-mediated signaling pathway is involved in both susceptibility and pathogenesis during *T. canis *infection in susceptible BALB/c mice.

**Figure 3:**
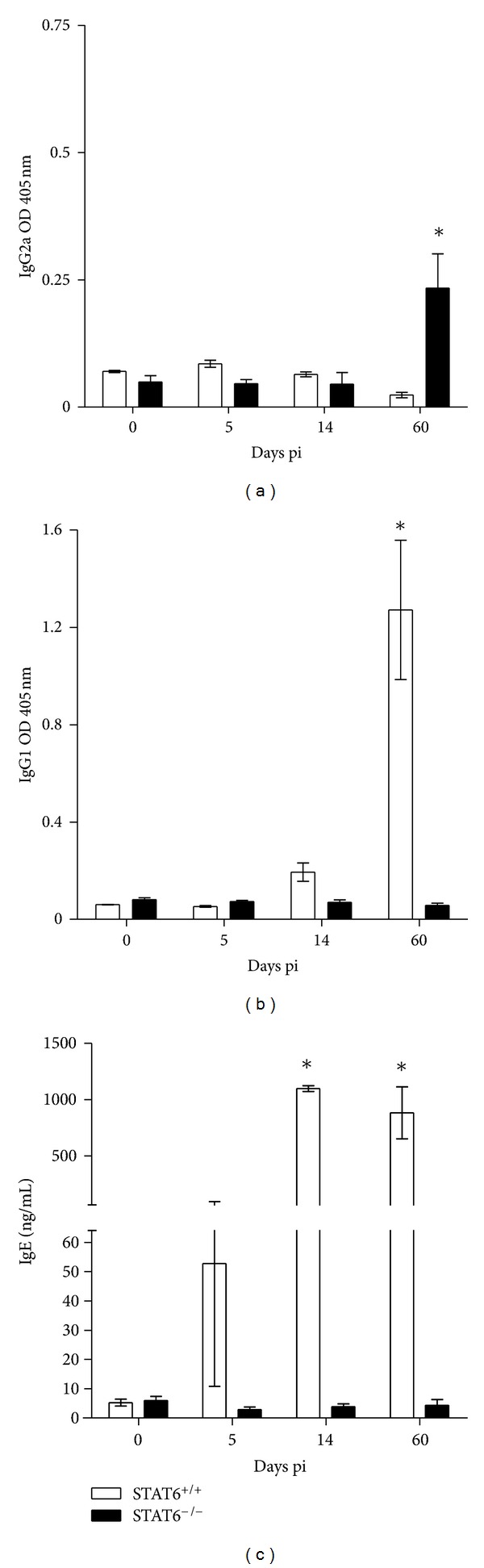
Kinetics of antibody production during *T. canis* infection by STAT6^−/−^ and STAT6^+/+^ mice. (a) Anti-*T. canis* specific IgG2a. (b) Anti-*T. canis* specific IgG1 and (c) Total IgE. Sera were taken from the vein tail of each mouse at time points described. ELISA plates were sensitized with 1 *μ*g/well of soluble extract of *T. canis*. The graphs show the mean ± SE (*n* = 4–6 animals) and are representative of two independent experiments. *P < 0.05 comparing STAT6^−/−^ versus STAT6^+/+^ at the same time point.

Previous studies have demonstrated that STAT6-mediated signaling pathway prevents development of protective immunity mainly against intracellular parasites by inhibiting Th1 development [25, 26]. Therefore, we measured levels of Th1-associated IgG2a as well as Th2-associated IgG1 and Total IgE antibodies in STAT6^−/−^ and STAT6^+/+^ mice at different time points following infection with *T. canis*. Additionally, we also compared the cytokine circulating levels from these same mice. Early in the infection, *T. canis*-infected STAT6^+/+^ and STAT6^−/−^ mice displayed minimum and comparable levels of *T. canis* Ag-specific Th1-associated IgG2a antibodies (Figure 3(a)); these data are in line with those previously reported [27], who also found a low production of this subclass of antibody at early times postinfection. However, by day 60 pi STAT6^−/−^ mice displayed significantly higher levels of specific IgG2a antibodies against *Toxocara* antigens (Figure 3(a)). On the other hand, clear differences were observed with the Th2-associated IgG1 production, where STAT6^+/+^ mice displayed significantly higher titers of *Toxocara*-specific IgG1 as compared to STAT6^−/−^ mice since day 14 after infection (Figure 3(b)). Although Th2-associated IgE has been shown to play a role in mediating immunity against certain extraintestinal helminthes, we found that *T. canis*-infected STAT6^−/−^ mice harbored lower parasite burdens despite producing significantly lower levels of IgE as compared to similarly infected STAT6^+/+^ mice, suggesting that IgE may have a limited role in mediating protective immunity against L2 of *T. canis* (Figure 3(c)). Here it is noteworthy that in spite of a higher Th2-associated antibody response in WT mice, these displayed greater susceptibility to *T. canis*. These data agree with those recently reported by [18], who found that after *T. canis* reinfection in BALB/c mice, reinfected mice displayed significantly higher titers of *T. canis*-specific IgG1; moreover, those antibodies showed a greater avidity for *T. canis* antigens. However, re-infected mice displayed a major number of larvae in different tissues [18]. Together with our data, such findings strongly suggest that a humoral immune response is not protective against L2 *T. canis* infection.

**Figure 4:**
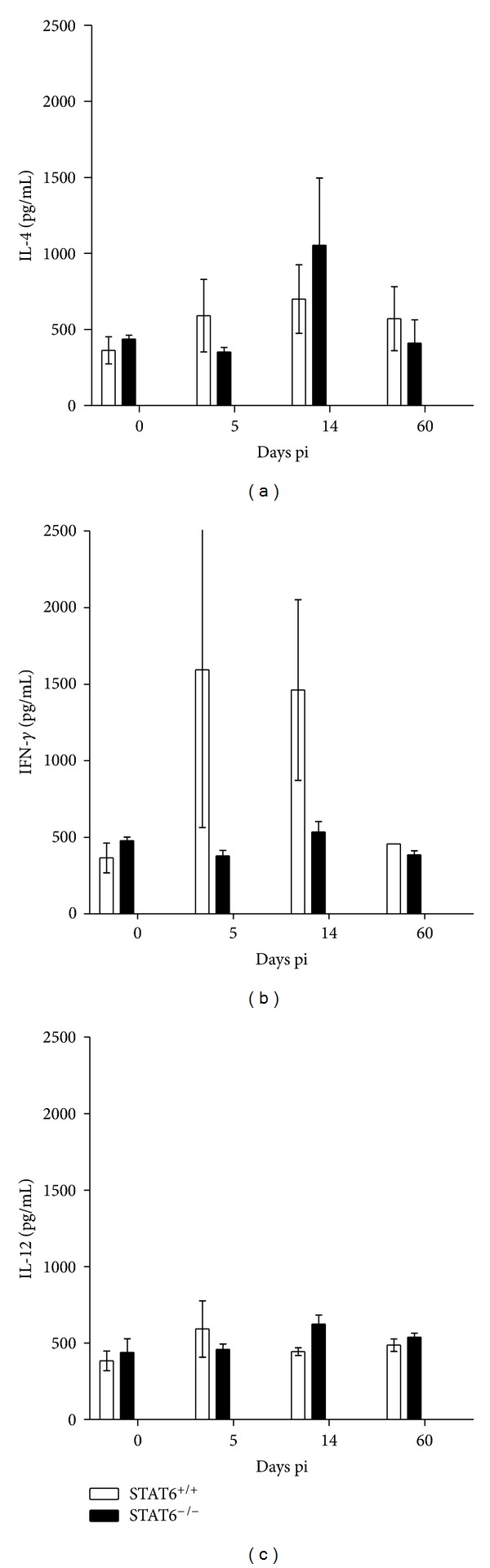
Cytokine profiles from the sera of STAT6^−/−^ and STAT6^+/+^ *T. canis-*infected mice. (a) IL-4 detection. (b) IFN-*γ* production and (c) IL-12 detection. Data are expressed as the mean ± SE and are representative of three independent experiments, *n* = 4. *P < 0.05 comparing STAT6^−/−^ versus STAT6^+/+^ at the same time point.

Next we analyzed the cytokine profile in sera that STAT6^+/+^ and STAT6^−/−^ mice displayed during toxocariasis. BALB/c mice at 5 dpi produced significantly more IL-4 and IFN-*γ* than STAT6^−/−^ mice (Figures 4(a) and 4(b)), whereas the levels of IL-12 were closely similar between groups (Figure 4(c)), indicating that not a clear Th1-type polarization of the immune response was observed in *T. canis*-infected STAT6^−/−^ mice. However, a lack of Th2-type response was confirmed in such mice given the low levels of IgG1 and IgE together with lower systemic levels of IL-4. 

**Figure 5:**
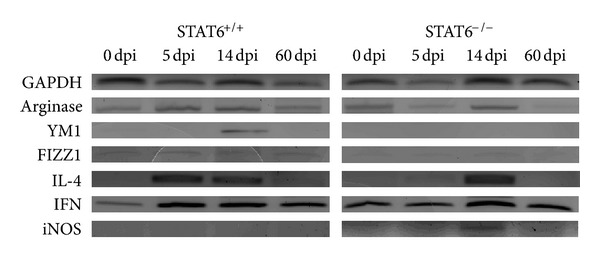
Spleen cells from STAT6^−/−^ and STAT6^+/+^ *T. canis*-infected mice display different levels of transcripts. Spleen cells were harvested at different times after infection and transcript levels of GAPDH, Arginase 1, Ym1, Fizz1, IL-4, IFN-*γ*, and iNOS were analyzed by RT-PCR. The data shown are from a single mouse and are representative of the findings from three mice examined at each time point.

To further analyze the immune response, the spleen cells from *T. canis*-infected STAT6^+/+^ and STAT6^−/−^ mice were obtained for RT-PCR analysis of several markers for alternatively activated macrophages (AAM) and some cytokines. While splenocytes from STAT6^+/+^ mice displayed expression of Arginase-1 and Ym-1, both markers for AAM, during early phase of infection, those from STAT6^−/−^ mice did not overexpress such markers (Figure 5(a)). In contrast STAT6^−/−^-infected mice displayed expression of iNOS, a marker for classically activated macrophages, at 14 dpi, whereas STAT6^+/+^ mice did not express iNOS (Figure 5(b)). Regarding cytokines we observed a major expression of IL-4 on spleen cells from STAT6^+/+^ than spleen cells from STAT6^−/−^ mice (Figure 5(a)), whereas a similar level of expression was observed for IFN-*γ* (Figure 5). Thus our data revealed the presence of AAM and IL-4 in the spleens of STAT6^+/+^ mice. 

In the last few years, a new cell population has been detected in most of the helminth infections, such population is AAM; the role of these cells appears to be divergent [30] as different authors have demonstrated, for example in gastrointestinal infections by *Nippostrongylus brasiliensis* and *Heligmosomoides bakeri* the presence of AAM is key for worm expulsion [31], whereas in other helminth infections such as *Trichuris muris* and *Hymenolepis diminuta* the presence of AAM was irrelevant [32–34]. In contrast, for schistosomiasis and experimental cysticercosis the presence of AAM appears to be crucial; in the first case, the absence of AAM leads to pathologic disorders in the liver and the hosts die [35, 36], whereas in experimental cysticercosis the presence of AAM leads to susceptibility, given that eliminating AAM with clodronate liposomes helps to clear the infection in otherwise susceptible hosts [37]. According to our knowledge this is the first time that the markers for AAM are reported in *T. canis* infection, but their role is still unknown. 

**Figure 6:**
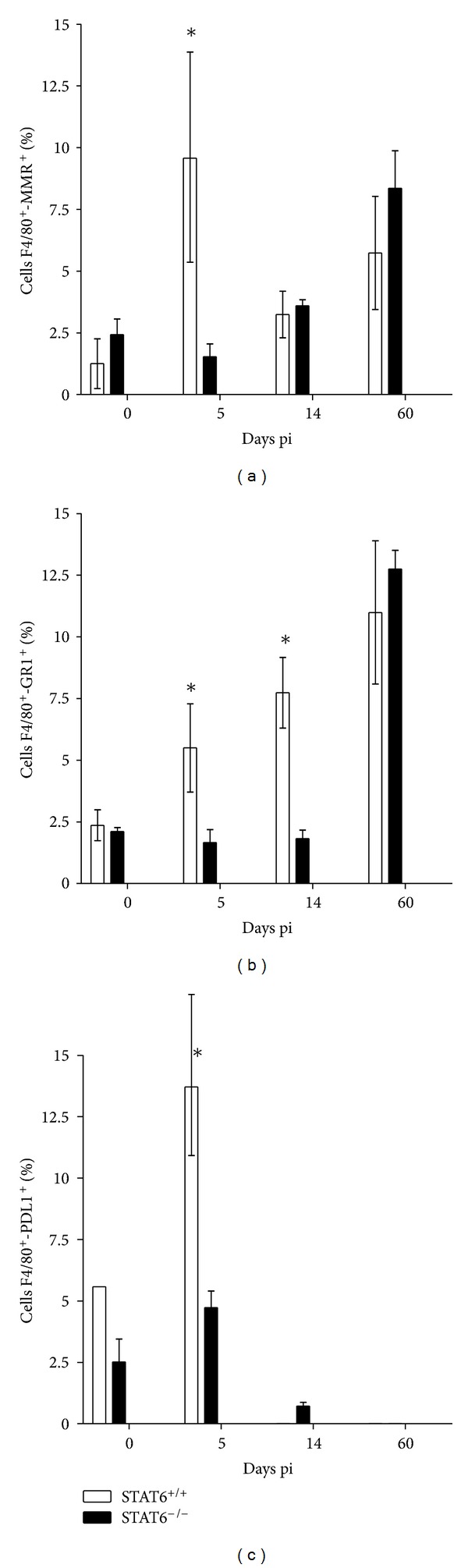
Lung macrophages from STAT6^−/−^ and STAT6^+/+^ *T. canis*-infected mice display different phenotypes. Macrophages were obtained at different time points after infection and analyzed by flow cytometry for the detection of different cell markers associated with alternative activation of macrophages. (a) F4/80^+^MR^+^ lung cells, (b) F4/80^+^Gr1^+^ lung cells, and (c) F4/80^+^PD-L1^+^ lung cells, as described in Section 2. Data are expressed as the mean ± SE and are representative of two independent experiments, *n* = 4. *P < 0.05 comparing STAT6^−/−^ versus STAT6^+/+^ at the same time point. ND: not determined.

In order to gain knowledge on a possible role for AAM in acute *T. canis* infection, we analyzed the profile of macrophages that reach the lungs as early as 5 days pi (time where striking differences in pathology were observed). After 5 dpi lungs were obtained and cut in small pieces, which were further passed through a mesh. Cells were stained for different markers and assayed for cytometry. Stained cells were captured in log, and the region that displayed both high granularity and high size was selected for analysis. As observed in Figure 6(a), lung cells from *T. canis*-infected STAT6^+/+^ mice displayed a greater recruitment of F4/80^+^MR^+^ cells compared to both naïve mice or *T. canis*-infected STAT6^−/−^ mice; these data suggest a recruitment of AAM, which may participate in tissue repair in the lungs, while in STAT6^−/−^ mice the increase of F4/80^+^MR^+^ cells was gradual, perhaps because they are unable to mount an efficient Th2 response that may impair the recruitment of AAM in the first days pi, which could be associated with a greatest tissue damage in the lungs (Figure 2(a)). Interestingly, by day 60 pi this population is increased in both groups, but still more damage prevailed in STAT6^−/−^ mice; this apparent contradiction may be explained just in a time-dependent point of view; it means that AAM early recruited in WT mice had more time to repair the tissue. In contrast, the apparent delay of AAM in reaching the lungs in STAT6^−/−^ mice may need longer time to accomplish their function. An alternate explanation may be the participation of different cell populations for a rapid tissue repair, thus, our observation that another population of cells F4/80^+^-Gr-1^+^ is also recruited differentially in the lungs of STAT6^+/+^ mice may be indicative that more than one-cell population is involved in tissue repair in this infection (Figure 6(b)). Intriguingly, such F4/80^+^-Gr-1^+^ took longer to reach the lungs in STAT6^−/−^ mice. However, these cells reach similar levels than in wild-type mice until 60 dpi (Figure 6(b)), if these cells represent a different population of regulatory cells needs further research. On the other hand, the same pattern of recruitment was followed when we analyzed the F4/80^+^PDL-1^+^ population (Figure 6(c)). All these surface markers have been previously reported associated with macrophages that undergo a distinct activation phenotype in the presence of the Th2 cytokines or helminth infections named AAM [38]. Besides, these AAM have an upregulated expression of arginase-1, RELM-*α*, and chitinase-like protein Ym1, among other markers. Moreover, although AAM can exhibit antiparasitic activity [31], their most important function in the context of migrating helminth parasites appears to be associated with tissue-repair responses [39, 40]. Taken together these cytometry analyses with those obtained in the macroscopic and microscopic lung analyses, we may associate less tissue damage on *T. canis-*infected STAT6^+/+^ mice with the presence of AAM, and by contrary, we would associate the absence of AAM in *T. canis*-infected STAT6^−/−^ mice with greater lesions and increased lung-cell infiltration. Therefore, we hypothesized that AAM may be involved in mediating protection against helminth-induced immunopathology in the lungs during acute toxocariasis.

 Several different reports have shown that Th2 responses are not definitive to kill extraintestinal phases of helminth parasites. For example during muscle infection with *T. spiralis*, BALB/c mice lacking eosinophils displayed similar larval burdens to those of wild-type BALB/c mice [21]. In line with our results, also *T. crassiceps* infection is fully controlled in the absence of STAT6 [22], and more interestingly during experimental neurocysticercosis caused by *Mesocestoides corti* infection STAT6^−/−^ mice displayed a reduction in the number of brain larvae but an increase in clinic neurological symptoms that were associated with lack of AAM [41]. Together all these findings oppose the dogma that Th2-type responses play a critical role in the elimination of all kind of helminthes [42]. These data also suggest that STAT6 pathway may act to limit *Toxocara* larvae-induced immunopathology at least in the lungs.

In conclusion, STAT6^−/−^ BALB/c mice mount a null Th2-like response and efficiently control *T. canis* infection. In contrast, STAT6^+/+^ BALB/c mice develop a predominant Th2-like response that is associated with high levels of IL-4, IgG1, IgE, and AAM and displayed significantly higher parasite burdens in different tissues but interestingly less associated pathology. The findings in our study support the conclusion that STAT6-mediated signaling is critical for the suppression of the immune response that is required for controlling L2 toxocariasis. We postulate that Th2 cytokines may have a dual role during toxocariasis, on one hand may contribute to host susceptibility via STAT6 activation and that neither AAM nor IgE are essential or primarily responsible for eliminating *T. canis *tissue infection, but on the other hand, such response may downregulate the immunopathology induced by *T. canis* larvae in the lungs. 

## References

[1] Avcioglu H, Balkaya I (2011). The relationship of public park accessibility to dogs to the presence of Toxocara species ova in the soil. *Vector-Borne and Zoonotic Diseases*.

[2] Dattoli VCC, Freire SM, Mendonça LR, Santos PC, Meyer R, Alcantara-Neves NM (2011). *Toxocara canis* infection is associated with eosinophilia and total IgE in blood donors from a large Brazilian centre. *Tropical Medicine and International Health*.

[3] Congdon P, Lloyd P (2011). Toxocara infection in the United States: the relevance of poverty, geography and demography as risk factors, and implications for estimating county prevalence. *International Journal of Public Health*.

[4] Pinelli E, Herremans T, Harms MG, Hoek D, Kortbeek LM (2011). Toxocara and Ascaris seropositivity among patients suspected of visceral and ocular larva migrans in the Netherlands: trends from 1998 to 2009. *European Journal of Clinical Microbiology and Infectious Diseases*.

[5] Walsh MG (2011). Toxocara infection and diminished lung function in a nationally representative sample from the United States population. *International Journal for Parasitology*.

[6] Amaral HLDC, Rassier GL, Pepe MS (2010). Presence of *Toxocara canis* eggs on the hair of dogs: a risk factor for Visceral Larva Migrans. *Veterinary Parasitology*.

[7] El-Tras WF, Holt HR, Tayel AA (2011). Risk of *Toxocara canis* eggs in stray and domestic dog hair in Egypt. *Veterinary Parasitology*.

[8] Jabbour RA, Kanj SS, Sawaya RA, Awar GN, Hourani MH, Atweh SF (2011). *Toxocara canis* myelitis: clinical features, magnetic resonance imaging (MRI) findings, and treatment outcome in 17 patients. *Medicine*.

[9] Hamilton CM, Brandes S, Holland CV, Pinelli E (2008). Cytokine expression in the brains of *Toxocara canis*-infected mice. *Parasite Immunology*.

[10] Finkelman FD, Shea-Donohue T, Goldhill J (1997). Cytokine regulation of host defense against parasitic gastrointestinal nematodes: lessons from studies with rodent models. *Annual Review of Immunology*.

[11] Gause WC, Urban JF, Stadecker MJ (2003). The immune response to parasitic helminths: insights from murine models. *Trends in Immunology*.

[12] Morimoto M, Zhao A, Sun R (2009). IL-13 receptor *α*2 regulates the immune and functional response to Nippostrongylus brasiliensis infection. *Journal of Immunology*.

[13] Urban JF, Maliszewski CR, Madden KB, Katona IM, Finkelman FD (1995). IL-4 treatment can cure established gastrointestinal nematode infections in immunocompetent and immunodeficient mice. *Journal of Immunology*.

[14] Perrigoue JG, Marshall FA, Artis D (2008). On the hunt for helminths: innate immune cells in the recognition and response to helminth parasites. *Cellular Microbiology*.

[15] Knott ML, Matthaei KI, Foster PS, Dent LA (2009). The roles of eotaxin and the STAT6 signalling pathway in eosinophil recruitment and host resistance to the nematodes Nippostrongylus brasiliensis and Heligmosomoides bakeri. *Molecular Immunology*.

[16] Finkelman FD, Shea-Donohue T, Morris SC (2004). Interleukin-4- and interleukin-13-mediated host protection against intestinal nematode parasites. *Immunological Reviews*.

[17] Urban JF, Noben-Trauth N, Donaldson DD (1998). IL-13, IL-4R*α*, and Stat6 are required for the expulsion of the gastrointestinal nematode parasite Nippostrongylus brasiliensis. *Immunity*.

[18] Kolbeková P, Větvička D, Svoboda J (2011). *Toxocara canis* larvae reinfecting BALB/c mice exhibit accelerated speed of migration to the host CNS. *Parasitology Research*.

[19] Othman AA, El-Shourbagy SH, Soliman RH (2011). Kinetics of Foxp3-expressing regulatory cells in experimental *Toxocara canis* infection. *Experimental Parasitology*.

[20] Terrazas LI, Bojalil R, Govezensky T, Larralde C (1998). Shift from an early protective TH1-type immune response to a late permissive TH2-type response in murine cysticercosis (*Taenia crassiceps*). *Journal of Parasitology*.

[21] Fabre V, Beiting DP, Bliss SK (2009). Eosinophil deficiency compromises parasite survival in chronic nematode infection. *Journal of Immunology*.

[22] Rodriguez-Sosa M, David JR, Bojalil R, Satoskar AR, Terrazas LI (2002). Cutting edge: susceptibility to the larval stage of the helminth parasite *Taenia crassiceps* is mediated by Th2 response induced via STAT6 signaling. *Journal of Immunology*.

[23] Takeda H, Tanaka T, Shi W (1996). Essential role of Stat6 in IL-4 signalling. *Nature*.

[24] Urban JF, Schopf L, Morris SC (2000). Stat6 signaling promotes protective immunity against Trichinella spiralis through a mast cell- and T cell-dependent mechanism. *Journal of Immunology*.

[25] Stamm LM, Räisänen-Sokolowski A, Okano M, Russell ME, David JR, Satoskar AR (1998). Mice with STAT6-targeted gene disruption develop a Th1 response and control cutaneous leishmaniasis. *Journal of Immunology*.

[26] Tarleton RL, Grusby MJ, Zhang L (2000). Increased susceptibility of stat4-deficient and enhanced resistance in stat6-deficient mice to infection with Trypanosoma cruzi. *Journal of Immunology*.

[27] Fenoy S, Rodero M, Pons E, Aguila C, Cuéllar C (2008). Follow-up of antibody avidity in BALB/c mice infected with *Toxocara canis*. *Parasitology*.

[28] Reyes JL, Espinoza-Jiménez AF, González MI, Verdin L, Terrazas LI (2011). *Taenia crassiceps* infection abrogates experimental autoimmune encephalomyelitis. *Cellular Immunology*.

[29] Khan WI, Vallance BA, Blennerhassett PA (2001). Critical role for signal transducer and activator of transcription factor 6 in mediating intestinal muscle hypercontractility and worm expulsion in Trichinella spiralis-infected mice. *Infection and Immunity*.

[30] McKay DM, Khan WI (2003). STAT-6 is an absolute requirement for murine rejection of Hymenolepis diminuta. *Journal of Parasitology*.

[31] Anthony RM, Urban JF, Alem F (2006). Memory TH2 cells induce alternatively activated macrophages to mediate protection against nematode parasites. *Nature Medicine*.

[32] Bowcutt R, Bell LV, Little M (2011). Arginase-1-expressing macrophages are dispensable for resistance to infection with the gastrointestinal helminth Trichuris muris. *Parasite Immunology*.

[33] Becerra-Diaz M, Valderrama-Carvajal H, Terrazas LI (2011). Signal transducers and activators of transcription (STAT) family members in helminth infections. *International Journal of Biological Sciences*.

[34] Johnston MJG, Wang A, Catarino MED (2010). Extracts of the rat tapeworm, Hymenolepis diminuta, suppress macrophage activation in vitro and alleviate chemically induced colitis in mice. *Infection and Immunity*.

[35] Herbert DR, Orekov T, Roloson A (2010). Arginase I suppresses IL-12/IL-23p40-driven intestinal inflammation during acute schistosomiasis. *Journal of Immunology*.

[36] Herbert DR, Hölscher C, Mohrs M (2004). Alternative macrophage activation is essential for survival during schistosomiasis and downmodulates T helper 1 responses and immunopathology. *Immunity*.

[37] Reyes JL, Terrazas CA, Alonso-Trujillo J, van Rooijen N, Satoskar AR, Terrazas LI (2010). Early removal of alternatively activated macrophages leads to *Taenia crassiceps* cysticercosis clearance in vivo. *International Journal for Parasitology*.

[38] Reyes JL, Terrazas LI (2007). The divergent roles of alternatively activated macrophages in helminthic infections. *Parasite Immunology*.

[39] Allen JE, Maizels RM (2011). Diversity and dialogue in immunity to helminths. *Nature Reviews Immunology*.

[40] Jenkins SJ, Allen JE (2010). Similarity and diversity in macrophage activation by nematodes, trematodes, and cestodes. *Journal of Biomedicine & Biotechnology*.

[41] Mishra BB, Gundra UM, Teale JM (2011). STAT6^−/−^ mice exhibit decreased cells with alternatively activated macrophage phenotypes and enhanced disease severity in murine neurocysticercosis. *Journal of Neuroimmunology*.

[42] Maizels RM, Hewitson JP, Smith KA (2012). Susceptibility and immunity to helminth parasites. *Current Opinion in Immunology*.

